# The Hospital for Sick Children, Great Ormond St.

**Published:** 1911-03-18

**Authors:** 


### THE HOSPITAL FOR SICK CHILDREN, GREAT ORMOND STREET.

### SPECIAL COURT OF GOVERNORS.

A Special Meeting of the Court of Governors of this Hospital was held on Wednesday, March 8, 1911, to answer certain charges recently made against the institution. There was a very full meeting, and Lord Peel was elected to the chair.

The Chairman said the purpose of the meeting was to answer the charges which had been specifically and definitely made against the administration of the hospital, and against the officers and staff of it. It was, of course, very easy to make charges and very difficult to relate them. But he was sure that meeting would give the case calm consideration after hearing the report of the Committee of Management, and then arrive at a decision on the matter.

The Secretary read the following report :

### The Report of the Committee.

On October 29, 1910, a paper called *Mrs. Bull* published an article headed "Great Ormond Street Children's Hospital—Shocking Disclosures—Little Children Infected with Foul Disease—Lame Excuse of the Authorities." The same paper has subsequently published further articles reflecting on the treatment of patients by the staff of the hospital.

An article answering the charges from a professional point of view was published in the *Lancet* on November 5, 1910. The editor had been requested to look into the matter, and, if he thought fit, to write an article. The article itself was not "inspired" by the committee or any member of the staff. The committee of the hospital also invited King Edward's Hospital Fund to make an inquiry into the matter. No further action has been taken. As the attacks continued, it has been thought right that the Governors at large should be taken into the confidence of the committee.

*John Bull* attributes the silence of the committee to indifference; need we say this is untrue? We have felt acutely accusations of nothing short of callous neglect, and even active cruelty, to the children. Especially have we felt these accusations brought against a staff of doctors, sisters and nurses who have devoted, and are devoting, their lives to the relief of suffering. It is, in fact, more for the sake of this staff than of ourselves that we have at last felt compelled to break silence. The original article charges against the authorities of the hospital that two of its patients became infected with a disease, which is there referred to as "disgusting and usually disgraceful." That infectious disease of all kinds should from time to time break out in a hospital is, of course, inevitable; but the article suggests that in the cases cited, it resulted from the incompetence of the staff and was a gross example of hospital blundering. This is not true. The disease (infective vaginitis) is very common among female children of the urban population, even young infants; it appears to be on the increase, or perhaps it would be more accurate to say that its presence is, owing to scientific methods, more frequently detected; in hospitals it must be considered one of the most insidious and most troublesome of house infections. It is intermittent, that is to say, a child may be apparently cured, but the symptoms may recur without any fresh cause of infection. It is communicable from patient to patient even during the intervals above referred to. It is highly probable, though perhaps not certain, that during the period of incubation also—from ten days to three weeks—it is contagious. Both these facts facilitate the spread of infection, even where every precaution is taken. It can generally be traced to some child admitted with an acute form of it; before the condition is, or, in many cases, can be, recognised and the patient quarantined, an entire ward may be infected and a local epidemic, which may last for months, may be the result. It is possible that, as is the case with some other diseases, a child may be a carrier of it though not actually suffering. In the case of young children the disease is seldom acquired by direct contact, but in such an institution as a children's hospital there are many channels by which it may be conveyed in spite of the most careful precautions. In the case of young children the disease is not, in itself, of a serious nature, and evil consequences in after life need not be apprehended. Another fact of the utmost importance is that a discharge, which is the visible symptom of the disease, does not necessarily indicate its presence. This can only be ascertained by bacteriological examination, and even by this means the micro-organism which causes the trouble cannot always be identified at once though actually present. Thus, two children exhibiting the same outward symptoms may be the one harmless, the other a source of danger to an entire ward. If a child is brought to the hospital for admission, and any discharge is observed—it is always looked for—the child is not admitted unless on bacteriological examination the specific organism is not discovered, or the child is suffering from some other disease rendering admission urgent. In that event the child is treated as a possible source of infection, and either sent to the isolation ward or so dealt with in the ordinary ward as to render contact, direct or indirect, impossible, or as difficult as may be. As has been stated above, however, a patient is sometimes admitted without the existence of the disease being in any way discoverable. If a patient in the hospital is found to have the disease, she is dealt with in the manner mentioned above in reference to fresh patients. One channel of contagion might be washing materials. These are kept separate for every patient in the hospital. In one of the articles it is stated that sponges are used for the patients indiscriminately, and that the water in the baths is not changed for each child. This is false. Flannels are used, never sponges, and the flannels are burnt on the departure of the patient for whom they have been used. Bath water is freshly drawn for every patient. It is possible, of course, that some precaution may have been omitted or some mistake made by a medical officer, sister, or nurse, but after careful investigation we are unable to discover any evidence that, with the possible exception of a case at Cromwell House, infection has in any case been due to such an error or to neglect.

As to the cases specifically referred to in that article. They have both been identified. As to the first case: On her discharge from Cromwell House on March 17 the child was found to have the contagious disease, and was not sent home as stated in the article. She was sent to the isolation ward and retained in hospital till June 1. She was then sent home, having been free from all outward symptoms for three weeks. She subsequently had a relapse and was readmitted on June 28 and was finally discharged on October 29, having then been free for more than a month.

The other child was sent home on March 17 and re-admitted on the 21st. She remained in the hospital until December 16, when her parents took her away.

With regard to the allegation that the children were sent out with dirty heads, a large proportion of children on admission are found to have parasites in their hair. The children's heads are brushed and tooth-combed every morning by probationers and examined twice a week by the staff nurse or sister. Enquiries have been made and there is no reasonable doubt that this routine was observed in the case of the children in question.

A second article appeared on November 12. It mentions two cases. The first has been identified as that of S. H. It is given under sensational headlines—"Kept waiting 3½ hours." "Refused admission." "Give me some water." "Dead." As to the first of these, the fact is, that the child was kept because he had not recovered from the effect of the operation. There was nothing unusual in this. A competent nurse was at hand, and there was no necessity for the services of a doctor. The parents were informed that the vomiting of swallowed blood referred to in the article might take place, and it is, in fact, not uncommon after such operations.

"*Refused admission.*"—The delay in admitting the child was due to a doubt on the part of the medical officers whether the case was infectious, and would therefore be a source of danger.

There is no justification for the charge implied in this headline, "Give me some water." If this and the paragraph it heads have any meaning, they convey a charge of gross cruelty in refusing to give even a glass of water to a suffering child. The fact is, that the refusal of the nurse to give the child drink at that time was in accordance with the instructions given to her by the medical officer in the interests of the child, who was suffering from incessant vomiting.

"*Dead.*"—The parents were warned on the Sunday that the child was seriously ill. His condition became suddenly critical in the evening of the Tuesday. When this was realised, a telegram was despatched to the father at 6.55. At 7.25 the child died, and the father arrived at 7.45. It is untrue, then, that no word was sent to the parents—the real sting in this paragraph. The committee fail altogether to see any just cause of complaint in this case. It is an instance of the way in which a journalist anxious for a sensation works one up out of the most ordinary facts. No enquiry as to this case was made of the hospital authorities before publication.

The other case is identified as that of W. T. It is only another instance of the class referred to in the first article, and is sufficiently dealt with in the earlier part of this report. The third article appeared on November 26. This is in the main a criticism on the article in the *Lancet*, and so far calls for no remark, except that the article is referred to as "inspired," which is not the fact. It is, however, suggested that the child (J. O.) was discharged because the parents had complained to *Mrs. Bull.* This is a gratuitous insult to the authorities of the hospital for which there is absolutely no ground. The child was discharged on October 29, having been free from the outward symptoms for more than a month." The report detailed the results of inquiry into other charges of a less grave nature, which showed that these accusations were grossly untrue, and then proceeded :

The case referred to as "another disgraceful disclosure" in the article of December 17 was the subject of an inquest, at which the relatives were represented by a solicitor. The coroner invited the jury, if they thought fit, to add to their verdict a censure on the hospital. They returned a verdict of "Death from natural causes," with no rider. The writer of the paragraph, the grandfather of the child, then laid the papers before the Committee of King Edward's Fund. Some of the members of that committee paid a surprise visit to the hospital. The committee thereafter made a few suggestions as to some matters of routine administration, but conveyed no censure on the staff as to the treatment of the case. The evidence at the inquest has been read and considered. We are satisfied that no act or omission on the part of the staff was proved which in any way contributed to the child's death, and the statements as to the operation are very inaccurate.

The article in *John Bull* of January 28 headed "Ghastly Hospital Horror" requires some notice. The facts are very simple : The child was admitted to the hospital when only one month old, although our usual practice is not to admit children under two years. He was brought by the grandmother, not by the mother as stated in the article. The practice of the hospital is to give to every person who brings a patient a printed leaflet containing the rules relating to in-patients, and this practice is strictly enforced. One of them is that "the child must be between two and twelve years of age." Another, printed in a conspicuous place in red ink, is as follows : "In the event of death a post-mortem examination will be made, unless objection should be made by the parent or guardian to the resident medical officer within twenty-four hours of the death or at the granting of the death certificate, if the same be granted within such twenty-four hours."

We have enquired of the nurse whose duty it was to attend to this matter, and we are satisfied that the leaflet is invariably given, and there is no reasonable doubt that it was given to the grandmother in this case. Whether she read or understood it is another matter. The rules apply to all in-patients, and to say that because that as to age is relaxed, the others do not apply, is a contention which answers itself.

The child died on December 10, a Saturday, at 8 P.M. On Monday, the 12th, the certificate of death was granted. No objection to a post-mortem examination was made. The cause of death stated on the certificate was "marasmus," which means simply, wasting. This was sufficient for the certificate, but what occasioned the wasting was not known. It was the grandmother who applied for the certificate. She had seen the body in the ward within half an hour of the death. The ward sister saw her on the 12th when she called; she did not ask then to see the body; if she had there would have been no objection. The porter denies making the remark attributed to him—in fact, he knew no more than that the child had died on Saturday evening, less than forty-eight hours before.

The body was decently coffined and was clothed in a gown provided by the ward sister herself.

The examination was made on the 13th. It was not a class demonstration : two of the members of the medical staff alone were present. Two incisions only were made, one across the top of the head, the other down the front of the body. No part of the body was retained. The examination, we are satisfied, was conducted with all decency and in the proper and ordinary manner. We have closely examined the port-mortem attendant, whose duty it is to sew up the body. He and the medical officers assure us that there is no truth in the details as to the tongue; this was in no way interfered with. As already mentioned, neither the brains nor anything else had been retained. The post-mortem was not wanted to get hold of the peritoneal or other glands for the purpose of examination as stated in the article.

### The Discussion of the Report.

Dr. Archibald Garrod, F.R.S. (senior physician to the hospital), said such charges would be found to be brought against any charitable institution in the land by some who felt that they were in some way aggrieved. One did not gather from those articles the opposite side of the picture: the infinitely greater number of parents who were satisfied with the treatment which was given to their children in the hospital. They did not hear of those who were more than satisfied, who were very grateful for what had been done for their children, and who wrote to the hospital occasionally to that effect, and some of whom volunteered the statement that they had written to the papers in question, but that their letters had not so far been published. (Applause.) He did not propose to refer to the charges in detail, nor did he wish to make light of them. Some of them were graver than others, but all of them proved, on investigation, to be based upon a one-sided statement. With regard to the more specific charges, that trouble was a very serious one in children's hospitals; the spread of infectious diseases was the chief trouble encountered in such hospitals. A familiar example, which most people experienced in their own homes, was measles. A child was admitted, not of course in health, but apparently not suffering from anything of that sort. After a week or so the child developed measles. The preliminary symptoms were very dubious and uncertain. But where there was the faintest suspicion of their nature, precautions were at once taken. If the development was more obvious, the child was removed, even before the rash had developed at all, to an isolation department, and in the majority of cases no spread occurred. Now and then a spread of measles took place, and the danger was so real that in some general hospitals no children's ward existed, because such would be regarded as a hotbed of infection. The particular trouble mentioned was notoriously a serious one in children's hospitals. The transference of infectious disease from one child to another could not be avoided by any known means compatible with the nursing of the patients in the wards. (Applause.)

Sir Thomas Barlow, K.C.V.O., submitted the following resolution: "That having heard the report of the Committee, and the statement of the Senior Physician, this Court of Governors desires to express its full confidence in the Committee of Management, in the honorary and resident medical staff, and in the nursing staff of the hospital." The disease mentioned in the articles was referred to as if it was a very terrible and exceptional condition, but it was known years ago at the hospital as a very common disease. He wished to lay stress on the fact that in the out-patient department it was a well recognised disease. But it was a very obstinate disease, and lasted for months, proving very refractory to treatment. It was not, in his experience, attended with serious or permanent damage to the little girls suffering from it. The terms used about the disease in the publication were not only sensational, but untrue. He had not seen a child permanently damaged by it. In some of the cases there was an affection of the joints; and if one studied the report carefully it would be evident that one of the children did not contract it in the hospital, but was brought to the wards suffering from the discharge and from a joint affection, which got quite well. This inquiry had been a very costly affair, and the subscribers might possibly grumble, but no pains had been spared in treating children and in taking every precaution. He could scarcely trust himself to speak about the nurses. Nobody knew how those women had suffered from the attacks. They had given the most devoted attention to the work, and their work in a children's hospital was much more severe than the nursing in a general hospital; they had to be very patient, and were on their feet during the whole time of their duty. They had always done their best for the little ones under their charge. Yet some mothers recently, though they were leaving their little ones in charge of the nurses, were actually distributing copies of the abominable articles.

Sir Trevor Lawrence seconded the resolution, which was put and carried with one dissentient.

Mr. Edmund Owen moved: "This Court is of opinion that the making of an attack upon a respectable hospital in order to obtain advertisement is unworthy of respectable journalism." Dr. David Lees seconded the resolution, which was carried, two voting against.

Mr. Lucas (Chairman of the hospital) remarked that the case for the Committee had been eloquently stated by Sir Thomas Barlow, but he would like to amplify one or two of the points to which Dr. Garrod had alluded. At the commencement of the trouble last October, when he was on the Continent, Mr. John Murray, the deputy-chairman of the Committee, invited a representative of the paper to meet him. A meeting was held, at which two of the senior members of the medical staff were present, with Mr. Murray and the Secretary. The details were fully gone into, both scientific and administrative. The paper, however, thought fit to suppress all statistics, and when the articles were published—he used the word advisedly—as a garbled report, then it was that the Committee came to the decision not to take any more action, but to leave the papers severely alone. He was glad to hear that Sir Thomas Barlow approved of that decision. Personally, he had nothing to regret from what might be called the neglect of those papers. Two members of the medical committee were told off to watch each case, and the lay members of the committee took careful notes as to the administrative part. Eventually it was decided to call the present meeting, and a sub-committee was appointed and took evidence in every case which was possible, and the result of their labours was embodied in the report. He wished to give a vivid illustration from his own experience as a result of the articles. A woman wrote to the matron at the hospital saying that unless she received money compensation because her child had had diphtheria in the hospital, she would at once communicate with the papers to which reference had been made. On receipt of that letter, he at once instructed the Secretary to tell the woman to come to the hospital. She came, but she would accept nothing as true which was stated to her by any doctor or any nurse, and she repeated the demand for money compensation for the trouble—a trouble with which, of course, one sympathised—she had had. That was not a singular instance; there were others of the same kind. The hospital did not fear criticism; it invited it, but it must be honest criticism. And he said, with full appreciation of the responsibility, that those criticisms had not been honest; they had been *ex-parte* statements without due inquiry, made by people who did not know hospital work, nor appreciate the grave injury which they were inflicting upon the hospital. It was difficult for the chairman of a hospital so attacked to exercise due self-restraint when speaking of such charges, but all who knew the inner working, the tender, loving care which was exercised by all concerned, would condemn, as that meeting had already condemned, such a wicked libel on the institution. He concluded by proposing a cordial vote of thanks to Lord Peel for presiding; this was carried by acclamation.

